# Burnout and resilience among intensive care workers facing the end of the COVID-19 pandemic: a cross-sectional study

**DOI:** 10.1590/1516-3180.2024.0244.R1.07032025

**Published:** 2025-08-11

**Authors:** Ana Irene Carlos de Medeiros, Elizabeth De Francisco Daher, Xinaida Taligare Vasconcelos Lima, Antonio George de Matos Cavalcante, Marcelo Alcântara Holanda, Eanes Delgado Pereira

**Affiliations:** I Professor, Departamento de Medicina Interna, Universidade Federal do Ceará (UFC), Fortaleza (CE), Brazil.; II Professor, Departamento de Medicina Interna, Universidade Federal do Ceará (UFC), Fortaleza (CE), Brazil.; III Professor, Departamento de Medicina Interna, Universidade Federal do Ceará (UFC), Fortaleza (CE), Brazil.; IV Professor, Departamento de Medicina Interna, Universidade Federal do Ceará (UFC), Fortaleza (CE), Brazil.; V Professor, Departamento de Medicina Interna, Universidade Federal do Ceará (UFC), Fortaleza (CE), Brazil.; VI Professor, Departamento de Medicina Interna, Universidade Federal do Ceará (UFC), Fortaleza (CE), Brazil.

**Keywords:** Burnout, professional, Resilience, psychological, Intensive care units, Healthcare workers, COVID-19, Coronavirus pandemic, Well-being, Mental health, Emotional exhaustion, Depersonalization

## Abstract

**BACKGROUND:**

Burnout is a global problem, and resilience may support the well-being of healthcare workers (HCWs) in stressful conditions.

**OBJECTIVES:**

This study aimed to evaluate the association between burnout and resilience among HCWs in intensive care units (ICU) during the coronavirus disease 2019 (COVID-19) pandemic.

**DESIGN AND SETTING:**

A cross-sectional study was conducted in the ICU of four public hospitals in Fortaleza, Brazil.

**METHODS:**

A face-to-face survey was conducted among HCWs in the ICUs of four public hospitals in Fortaleza between January and August 2023. The participants completed questionnaires on burnout and resilience.

**RESULTS:**

A total of 194 professionals, including physichians (24%), nurses (29%), nursing technicians (25%), and physiotherapists (22%), completed questionnaires on burnout and resilience through face-toface interviews. Most professionals (62%) presented with overall burnout symptoms, and an inverse association was observed between resilience and burnout. However, 16 of the 44 (34%) HCWs with the highest resilience scores experienced burnout. Furthermore, younger age and higher workload were associated with a higher prevalence of burnout.

**CONCLUSION:**

Determinants of burnout were identified among ICU staff members during the last year of the COVID-19 pandemic. Resilience helped HCWs cope with burnout. However, some of the most resilient HCWs presented with high levels of burnout. Efforts are necessary to implement resilience-building tools, yet public health policies to improve ICU organizational issues are more important and urgent for promoting sustainable well-being among professionals, particularly during challenges such as pandemics. Introducing resilience-building tools and implementing public health policies are necessary to improve ICU management and promote sustainable well-being among healthcare workers in high workload settings.

## INTRODUCTION

 The escalating demands placed on healthcare systems during the coronavirus disease 2019 (COVID-19) pandemic have severely strained intensive care unit (ICU) healthcare workers (HCWs), especially younger professionals, those with less experience, and those with increased workloads.^1-3^ The global burnout epidemic has affected thousands of health professionals and has become a public health problem. In the face of this global pandemic, the World Health Organization recognized burnout as an occupational illness and incorporated it into the International Classification of diseases (ICD-11).^4^

 The inverse association between burnout and resilience was well documented in a national survey of healthcare professionals conducted prior to the pandemic.^5^ Another large-scale survey of ICU HCWs in 2021 demonstrated that higher levels of resilience were associated with reduced symptoms of anxiety, depression, and post-traumatic stress disorder.^6^ However, there is a scarcity of studies investigating the levels of burnout and association with resilience among ICU HCWs toward the end of the COVID-19 pandemic.^7^

## METHODS

### Study Design, setting, and participants

This survey was conducted in public adult ICUs in four tertiary hospitals in Fortaleza, Brazil, from January to August 2023. All HCWs, including physicians, nurses, nurse technicians, and physiotherapists signed a written consent form and answered questionnaires on burnout and resilience through face-to-face interviews. This study was approved by the National Research Ethics Committee (Protocol No. 04582818.6.00005054/2019).

### Questionnaires

 The Resilience Scale for Adults (RSA)^8,9^ is organized into six factors, and the scores are calculated using total values ranging from 33 to 231, with higher scores indicating higher levels of resilience. 

 The Maslach Burnout Inventory (MBI) contains three domains of burnout: emotional exhaustion, depersonalization, and professional efficacy. Consistent with the convention, we considered HCWs with a high score on the emotional exhaustion subscale (scores ≥ 26 on a 0–54 scale) and/or depersonalization subscale (scores ≥ 9 on a 0–30 scale) of the MBI to have at least one manifestation of professional burnout.^5,10^

### Statistics

 A previous study showed mean (SD) resilience of 178.17 ± 22.44 among HCWs.^11^ The z-score (z) was 1.96, the margin of error (e) was 0.05, and, assuming a similar mean with a 95% confidence interval (CI), 160 participants were required. 

 Standard descriptive statistics were used to describe healthcare worker characteristics, a chi-square test was used to compare resilience and burnout, and multivariable logistic regression was used to examine the association between resilience and burnout, adjusting for sex, age, occupation, weekly working hours, and employment at two or more hospitals. Independent variables were selected from the literature. Multicollinearity was assessed using the variance inflation factor (VIF) and values > 2 were excluded. All tests were two-sided with type I error rates of 0.05. All analyses were performed using IBM SPSS Statistics for Windows, version 26.0. 

## RESULTS

 In total, 194 HCWs participated in this cross-sectional study, including 46 physicians (24%), 56 nurses (29%), 49 nursing technicians (25%), and 43 physiotherapists (22%). Most patients were female (71%), and 50% were under 35 years of age. A total of 62% of the participants presented high levels of global burnout. Importantly, none of the respondents felt supported by their healthcare institution. 

 The mean (SD) resilience total score was 176.3 ± 28.2 and the median (Interquartile range 25%–75%) was 181(155-199) out of 231 scores. A total of 76% of HCWs with the 25th resilience score had burnout symptoms, and 34% of HCWs with the 75th resilience score had burnout symptoms (Table 1). 

**Table 1:** Emotional exhaustion, depersonalization, and overall burnout proportions across percentiles of resilience scores among healthcare workers

**Variables**	**25th (≤ 154 points) (n = 47)**	**25th–75th (155–199 points) n = 100**	**75th (≥ 200 points) n = 44**
Overall Burnout*	36 (76%)^#^	68 (68%)^ϯ^	16 (34%)
Exhaustion*	33 (70%)^§^	58 (58%)^Ϯ^	14 (31%)
Depersonalization*	29 (61.7%)^a,b^	36 (36%)^c^	7 (15.9%)

* Chi-square test and post hoc analysis for pairwise comparisons.

# 25th versus 75th (P < 0.05);

ϯ 25th–75th versus 75th (P < 0.05);

§ 25th versus 75th (0 < 0.05).

Ϯ 25th–75th versus 75th (P < 0.05);

a 25th versus 25th–75th (P < 0.05);

b 25th versus 75th (P < 0.05);

c 25th–75th versus 75th (P < 0.05).

 Multiple variable analysis revealed an inverse association between resilience and burnout. Each 1-point increase in resilience score was associated with lower odds of emotional exhaustion, depersonalization, and overall burnout symptoms [odds ratio (OR) 0.97; 95% CI (0.96–0.99), OR 0.97; 95% CI (0.96–0.99), and OR 0.97; 95% CI (0.96–0.98), respectively]. Working in more than two hospitals increased the odds of emotional exhaustion, depersonalization, and overall burnout symptoms [OR 2.48; 95% CI (1.20–5.11), OR 2.71; 95% CI (1.23–5.94), and OR 3.03; 95% CI (1.40–6.75), respectively]. HCW younger than 35 years of age had higher odds of emotional exhaustion and overall burnout symptoms [OR 2.25; 95% CI (1.06–4.77) and OR 3.32; 95% CI (1.12–4.82), respectively] (Table 2). 

**Table 2:** Multivariable logistic regression model of the association between resilience score and burnout symptoms among 194 healthcare workers

**Variables**		**Characteristics n = 194**	**High EE Unadjusted OR (95% CI)**	**High EE Adjusted OR (95% CI)**	**High DE Unadjusted OR (95% CI)**	**High DE Adjusted OR (95% CI)**	**High overall burnout Unadjusted OR (95% CI)**	**High overall burnout Adjusted OR (95% CI)**
**Gender**								
	*Female*	138 (71%)	reference	reference	reference	reference	reference	reference
	*Male*	56 (29%)	0.58 (0.30–1.11)	0.97 (0.43–2.26)	0.36 (0.19–0.69)	0.97 (0.21–1.14)	1.71 (0.87–3.36)	1.06 (0.46–2.53)
**Age in years**								
	*> 35*	97 (50%)	reference	reference	reference	reference	reference	reference
	*≤ 35*	97 (50%)	1.58 (0.89-2.81)	**2.25 (1.06–4.77)**	**2.16 (1.19–3.90)**	2.04 (0.93–4.50)	**2.14 (1.17–3.92)**	**3.32 (1.12–4.82)**
	*Working in more than two hospitals*	83 (42.8%)	**2.40 (1.33–4.34)**	**2.48 (1.20–5.11)**	**2.45 (1.35–4.46)**	**2.71 (1.23–5.94)**	**2.87 (1.53–5.38)**	**3.03 (1.40–6.75)**
	*Working More than 40 hours per week*	188 (96%)	1.83 (0.95–3.47)	1.13 (0.67–0.99)	0.77 (0.39–1.52)	0.77 (0.33–1.77)	2.12 (1.10–4.07)	1.63 (0.74–3.59)
**Profession**								
	*Physician*	46 (23.7%)	reference	reference	reference	reference	reference	reference
	*PT*	43 (22%)	**0.38 (0.16–0.90)**	0.53 (0.21–1.71)	**0.22 (0.08–0.59)**	0.48 (0.15–1.61)	**0.27 (0.11–0.68)**	0.43 (0.15–1.41)
	*Nurse*	56 (28.8)	1.05 (0.46–2.37)	1.46 (0.46–3.42)	1.15 (0.52–2.51)	2.21 (0.75 6.29)	1.05 (0.43–2.58)	1.82 (0.59–5.65)
	*Nurse technicians*	49 (25.2%)	0.51 (0.22–1.17)	1.06 (0.36–2.91)	**0.32 (0.13–0.77)**	0.50 (0.21–1.86)	**0.39 (0.16–0.94)**	0.93 (0.32–2.72)
**Resilience scores (mean ± SD)**		176.3 ± 28.2	**0.97 (0.96–0.98)**	**0.97 (0.96–0.99)**	**0.97 (0.96–0.98)**	**0.97 (0.96–0.99)**	**0.97 (0.96–0.98)**	**0.97 (0.96–0.98)**

Abbreviations: EE = emotional exhaustion; DE = depersonalization; OR = odds ratio; CI = confidence interval; PT = physiotherapist; SD = standard deviation.

## DISCUSSION

 This cross-sectional study demonstrated that HCWs in the ICUs of public hospitals remained vulnerable to burnout at the end of the COVID-19 pandemic. An association between higher resilience and less burnout symptoms was identified; however, some of the most resilient professionals did experience burnout. Other conditions such as increased workload and younger age were associated with a higher prevalence of burnout. Despite the high levels of burnout, healthcare professionals do not receive support from healthcare institutions. This highlights the need to invest in institutional policies to mitigate this problem. 

 The proportion of respondents with global burnout in a previous multicenter study,^3^ was closer to ours (51% versus 62%). In 2020, a survey conducted by our group in the same ICUs showed a similar prevalence of emotional exhaustion and depersonalization compared to data from 2023 (48% versus 54% and 29% versus 37%, respectively). This finding indicates that the HCWs continued to work under stressful conditions. 

 The association between workload, younger age, and burnout has been demonstrated by other studies.^1,12^ Employment at more than two hospitals is a modifiable factor that hospital managers should pay attention to. For example, job stability, good working conditions, and mental health support can help maintain a permanent professional workforce in hospitals. 

 Younger HCWs showed higher levels of burnout, and there was an imbalance between expectations and the reality of high-demand tasks in ICUs for this group of professionals. Encouraging partnerships between more experienced and younger healthcare professionals can help them. 

 Despite the small sample size, this study found an inverse relationship between burnout and resilience. 

## CONCLUSION

 This cross-sectional study of four public hospitals suggests that HCWs remain vulnerable to burnout even toward the end of the COVID-19 pandemic. This study also supports the clinical relevance of the identified modifiable factors of burnout that should be considered to mitigate stress in HCWs. Although resilience protects against burnout, some of the most resilient professionals presented burnout, suggesting that efforts to strengthen resilience are important. However, broader ICU system-level improvements are needed to promote sustainable well-being among healthcare workers, particularly during pandemics. 
